# Rewetting drained forested peatlands: A cornerstone of Sweden’s climate change mitigation strategy

**DOI:** 10.1007/s13280-025-02220-x

**Published:** 2025-07-16

**Authors:** Hjalmar Laudon, Järvi Järveoja, Anneli Ågren, Matthias Peichl, Amelie Lindgren

**Affiliations:** 1 Department of Forest Ecology and Management, Swedish University of Agricultural Sciences, 901 83 Umeå, Sweden; 2 Department of Earth Sciences, University of Gothenburg, 405 30 Gothenburg, Sweden

**Keywords:** Climate strategy, Greenhouse gases (GHG), LULUCF (land-use, land-use change and forestry), Peatlands, Rewetting

## Abstract

Peatland rewetting has developed into a key strategy to limit greenhouse gas (GHG) emissions, enhance carbon uptake, and restore biodiversity. With an increasing political ambition to enhance rewetting across many countries, there is a risk of prioritizing peatlands that are most readily available before the ones that result in the largest climate and biodiversity benefits. Based on the best current understanding, we provide a conceptual model of the climate impact and discuss some key steps of progress needed. We focus on Swedish conditions, but also use relevant studies from similar hydroclimatic conditions elsewhere. We argue that the large political interest and investments now made to rewet large areas of peatlands, in combinations with the many unknowns, make it more important than ever to start new rewetting research studies that includes various key aspects of GHG, hydrology, and biodiversity along large climate, land-use history, and nutrient gradients.

## Introduction

Centuries of drainage to increase both the land-area availability and plant production potential have affected a large fraction of the global peatlands with considerable greenhouse gas (GHG) consequences because of enhance mineralization of organic soils (Leifeld et al. 2019). Consequently, rewetting has become an important climate change mitigation strategy in many countries with the expectation that higher groundwater (GW) levels will reduce CO_2_ emissions from peat decomposition and that rewetted peatlands with time will return to becoming active carbon (C) sinks (Günther et al. 2020). Rewetting is therefore discussed as a key route to help reach the goal set out in the EU ‘Land-use, land-use change, and forestry’ (LULUCF) Directive (Regulation (EU) 2023/839 2023), which requires many EU countries to increase their GHG uptake by 2030 compared to 2016–2018. Rewetting is also included in the EU Nature Restoration Law (Regulation (EU) 2024/1991 2024) that sets binding targets for the area of drained organic soil restoration. However, key uncertainties still need to be addressed to optimize the effectiveness of this climate change mitigation strategy. This includes better understanding of where to focus restorations ambitions to maximize the climate benefit and how to improve the prediction of the time it takes to reach the expected climate cooling effect. Such uncertainty reductions require improved scientific assessments of knowns and unknowns regarding rewetting effects on the long-term GHG balance.

While high-latitude natural peatlands are one of the largest terrestrial C pools globally (Bradshaw and Warkentin 2015), a century of intensive drainage activities followed by a range of different land uses has reduced this large C stock due to peat mineralization (Krause et al. 2021; Lazdins et al. 2024). The mechanism behind the large build-up of soil organic matter (SOM) in peatlands is primarily driven by water-saturated soils and cold climate, providing conditions were plant remains decompose slowly. Hence, despite the generally low biomass production of most pristine peatlands—primarily through the growth of *Sphagnum* moss and sedges—plant detritus accumulation has been an ongoing process since the last glaciation (Ovenden 1990). While this SOM stock is of global significance, the balance between production and decomposition can easily be perturbed by hydrological alteration. Drainage of peat soils generally results in enhanced mineralization rates and therefore in increased net soil GHG emissions. In fact, the net GHG emissions from drained organic soils in Sweden have been estimated to be nearly equal to the emissions from the passenger car traffic in the country (Swedish Environmental Protection Agency 2024). To reduce national GHG emissions, peatland rewetting is now implemented in various projects across Sweden. However, the size of GHG emission reduction likely varies greatly depending on peatland type, past and current land use, vegetation succession, and time since rewetting (Ojanen and Minkkinen 2020; Laine et al. 2024).

Emissions from drained peat soils have been measured through empirical field and experimental studies (Jauhiainen et al. 2019), which have been collated into emission factors (EFs) aimed for national reporting of GHG emissions from drained organic soils (Hiraishi et al. 2014). In comparison, data for developing robust EFs for rewetted peatlands are scarce, specifically in the boreal region. Instead, EFs for rewetted boreal peatlands are primarily based on data from natural peatlands used as analogs. Furthermore, EFs for drained and rewetted peatlands are stratified into broad categories based on climate zone, current land use, and soil nutrient status, with no consideration of existing climate gradients or prior land use. Currently used EFs also do not reflect temporal dynamics of GHG emissions that occur in rewetted systems as they develop. Due to these limitations, EFs (both for drained and rewetted conditions) will not provide enough information to find optimal areas for rewetting. Hence, to help guide policy and peatland managers into best management practice (BMP), it is important to expand our knowledge of these systems through long-term monitoring schemes. These studies should include both GHG emissions and environmental data on GW levels, vegetation succession, and soil properties.

There are theoretical reasons to expect that rewetting with time will revert drained peatlands toward more naturally functional C accumulating systems (Ojanen and Minkkinen 2020), despite questions about whether or not they will resume all of the important ecological and hydrological functions of the pristine ecosystem (Kreyling et al. 2021). While rewetting will cause a switch from net source to net sink of C in most cases (Günther et al. 2020), several questions remain unanswered concerning net climate effects over time. Most of all, a robust empirical understanding of the climate impact of rewetting requires assessment of all relevant fluxes contributing to the full GHG balance. Compared to CO_2_, methane (CH_4_) and nitrous oxide (N_2_O) have global warming potentials (GWPs) that are 81 and 273 times greater over a 20-year period, respectively (IPCC 2021). Since CH_4_ has a relatively short atmospheric lifetime, its GWP is reduced to 27 over a timeframe of a century. Additionally, dissolved C losses through stream runoff must also be accounted for. At present, however, such holistic assessments are essentially lacking, at least at timescales that are long enough to become relevant. The omission of any of these key components might in the worst-case result in rewetting efforts that exacerbate rather than reduce net GHG emissions in the short term, particularly in systems with comparably low GHG emissions prior to rewetting. However, it is important to realize that drained peatlands that will not be rewetted in many cases likely will continue to be large soil CO_2_ emitters as long as the groundwater levels remain low. Hence, it can be argued that the faster the rewetting efforts are initiated the sooner will the soil C loss be reduced (Günther et al. 2020).

However, with the political ambitions of rapidly increasing the rate of rewetting across many countries, there is a risk of prioritizing peatland sites that are most readily available before the ones that would result in the largest climate benefits. This is especially problematic without a proper assessment of the current scientific understanding about where rewetting should be done to optimize the climate change mitigation potential. To help avoid such pitfalls, we discuss the current state of the art about rewetting effects and provide a conceptual model of the climate impact and present unresolved questions on the GHG balance of drained forest peatlands relevant for Swedish conditions with the goal to direct new studies and guide future rewetting ambitions.

## Rewetting as Sweden’s national strategy for climate mitigation

**Fig. 1:**
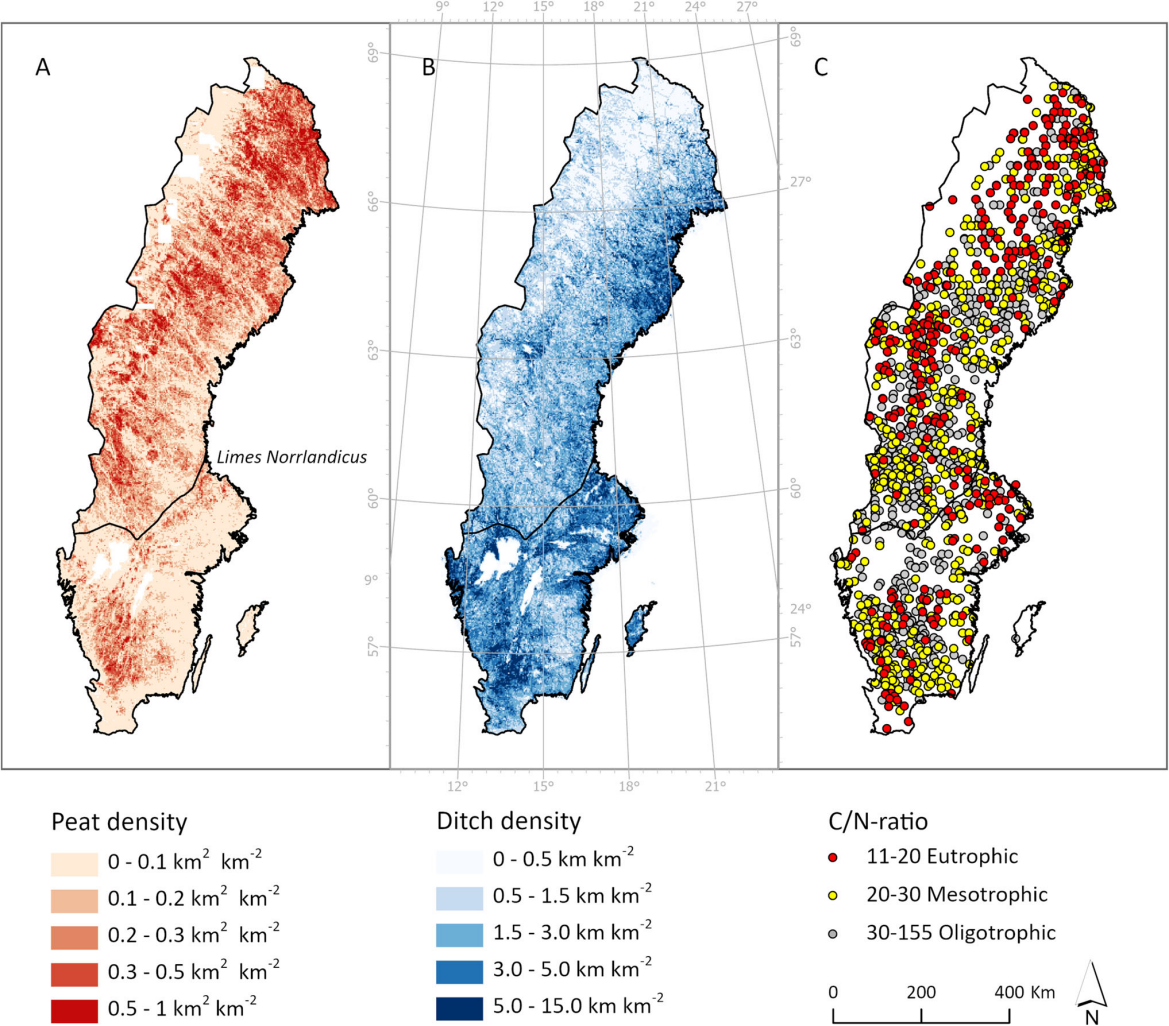
Maps over Sweden. **A** Distribution of peat ≥ 50 cm deep (km^2^ km^−2^) according to Ågren et al. (2022). **B** Distribution of ditches (km km^−2^) according to Lidberg et al. (2023). **C** C/N ratio in peatlands ≥ 50 cm deep (analyzed in samples 0–30 cm depth), where gray points indicate oligotrophic (nutrient-poor), yellow mesic (intermediate), and red eutrophic (nutrient-rich) sites

In a Swedish context, the potential of forest peatland rewetting is enormous. Decades of drainage in the early 1900s resulted in almost half a million km of ditches on organic soils, which may have affected several million ha of peatlands in the forested landscape (Fig. 1). With the current annual rate of ~ 1500 ha of peatlands being rewetted (Swedish Environmental Protection Agency (2024), it would take several thousands of years before all drained peatlands in Sweden would be rewetted. With this unprecedented scope of potential rewetting, it is imperative that science help design BMP in terms of the maximum gains of rewetting different types of peatlands.

The Swedish Forest Agency (SFA) suggested that the BMP for rewetting drained forested peatlands is to focus on nutrient-rich sites in the south of Sweden (Drott and Eriksson 2021). This recommendation builds on the current use of EFs (Hiraishi et al. 2014), which suggests that emissions from drained organic soils are higher in the temperate zone compared to the boreal. It has also been suggested that arable peatlands should be prioritized due to their low GW table and high nutrient status, which results in high soil CO_2_ and N_2_O emissions (Kasimir and Lindgren 2024). Further, it is generally assumed that most fertile forested peatlands occur in the southern parts of Sweden, but this is potentially a misconception. While the C/N ratio decreases toward the south on mineral forest soils (Högberg et al. 2021), data from the national soils inventory demonstrate that eutrophic peatland soils in the forested landscape are most common in the central and northern parts of the country (Fig. 1c, Table 1). To meet the Swedish national climate goals, efforts must be directed to areas where the rewetting has the greatest potential for emission reductions. While this clearly points toward organic soils in the agricultural landscape (Kasimir and Lindgren 2024), a substantial portion of these areas are dedicated to food production. Despite the explicit focus of the EU Nature Restoration Law on such soils, many may ultimately be unavailable for rewetting. Instead, abandoned arable land area across Sweden, often afforested in the last centuries (Swedish Board of Agriculture 2012), and other nutrient-rich forested peatlands (Table 1) are now instead of key interest for the bulk of potential rewetting actions in future.

**Table 1:** The percentage of each nutrient class of peatlands (defined as ≥ 50 cm deep peat) in the Swedish Forest Soil Inventory (SFSI) plots (from Fig. 3C). The peat cover in km^2^ from each area was calculated from the classified ≥ 50 cm deep peat map from Ågren et al. (2022). North and South Sweden is divided by Limes Norrlandicus (Fig. 3A)

	Nutrient class (%)	Peat cover ≥ 50 cm (km^2^)
*North Sweden, total*		57 060
Eutrophic (C/N ratio < 20)	22	12 508
Mesotrophic (C/N ratio 20–30)	33	18 588
Oligotropic (C/N ratio > 30)	46	25 964
*South Sweden, total*		11 205
Eutrophic (C/N ratio < 20)	15	1744
Mesotrophic (C/N ratio 20–30)	35	3914
Oligotropic (C/N ratio > 30)	50	5547

## Factors regulating greenhouse gas emissions and uptake

To date, relatively few studies have empirically investigated the climate change mitigation potential of peatland rewetting under boreal and hemiboreal conditions (but see Tong et al. (2025)). Those that do exist are limited to short-term warm season investigations of individual GHG’s (Komulainen et al. 1998, 1999; Laudon et al. 2023) or based on comparison between natural and drained peatlands (Laine et al. 2024). Assessing the net GHG balance is complex for both drained and rewetted systems and needs comprehensive field experimental studies (Jauhiainen et al. 2019). Each of the three key gases, CO_2_, CH_4_, and N_2_O, has different mechanistic regulation, and time-sensitive GWP. Also, the aquatic losses of organic (DOC) and inorganic carbon (DIC) via stream and ditch-networks merit more consideration.

**Fig. 2:**
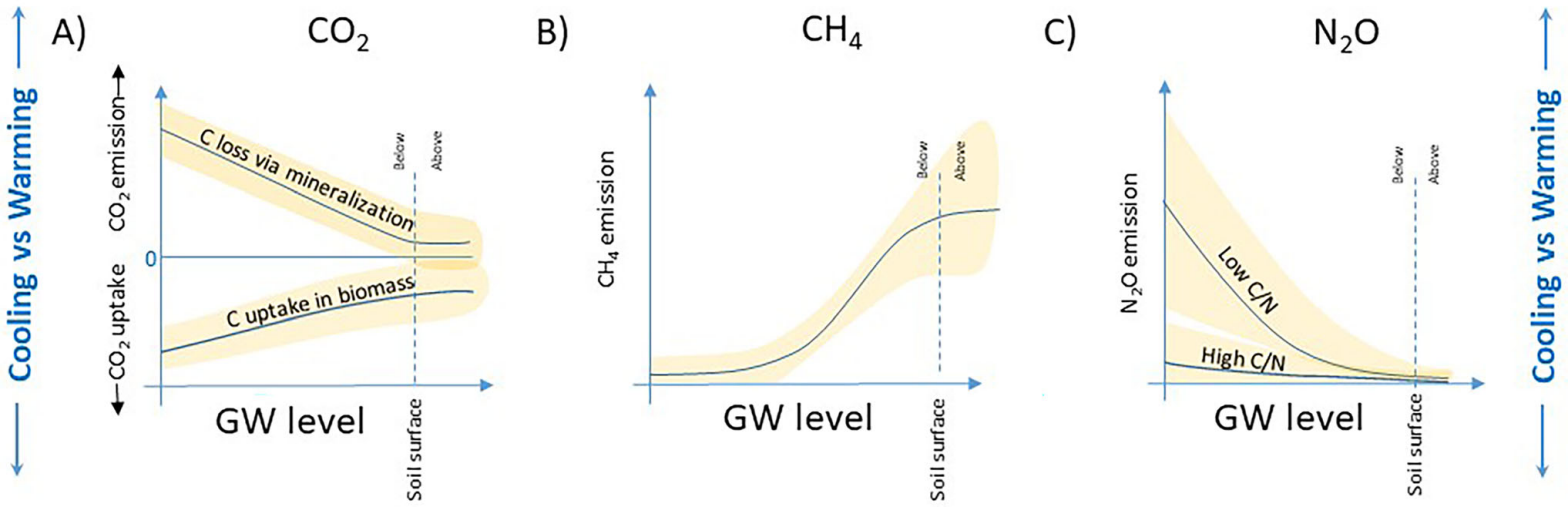
Groundwater (GW) level as a key regulator of greenhouse gas (GHG) emissions of carbon dioxide (CO_2_) (panel **A**), methane (CH_4_) (panel **B**), and nitrous oxide (N_2_O) (panel **C**). The yellow areas denote unexplained variability around the main effect

The net ecosystem CO_2_ balance of peatlands is regulated by the difference between plant growth that assimilate CO_2_ in biomass (i.e., net primary production—NPP) and the decomposition (mineralization) of organic matter returning CO_2_ to the atmosphere (i.e., heterotrophic respiration—Rh). The magnitude of both CO_2_ uptake and release is to a large extent regulated by the GW level (Fig. 2a). Lower GW levels enhance the CO_2_ emission rate because oxygenated conditions in unsaturated peat soils result in increased mineralization of SOM. However, lower GW levels may also enhance the plant growth rate, which increase the CO_2_ uptake potential because many plants that establish after drainage grow faster than most plants that dominate on peatlands. Hence, any impact affecting the GW level may perturb the two component fluxes, which determines the CO_2_ sink-source function of a peat soil (Meyer et al. 2013; He et al. 2016). The peat CO_2_ balance can thus change from a net uptake when GW is close to the soil surface (0–20 cm) and result in a sharp increase in CO_2_ emissions the deeper the GW level is located (Evans et al. 2021). Rewetting will thus result in a lower peat mineralization rate. However, rewetting of peatland forest also eliminates the C uptake via trees and may lead to greater litter production from decaying non-wet-adapted ground vegetation, which can result in enhanced CO_2_ emissions during the initial stages. The long-term enhanced net uptake can be expected as new peat forms as a result of the establishment of wet-adapted plants like *Sphagnum* mosses, graminoids, and some herbs.

The net balance of CH_4_ production and oxidation is to an even larger extent controlled by the GW level. In peatland soils, methanogens produce CH_4_ in the anaerobic zone, because of water saturation. Some of the CH_4_ can be oxidized to CO_2_ by methanotrophic bacteria in the upper aerobic soil layer. In practice, this balance between the production below the GW level and consumption above means that the highest CH_4_ emissions rates occur when the GW is close to or above the soil surface (Fig. 2b). However, across the boreal and hemiboreal parts of Europe and North America, only few published short-term studies exist from rewetted peatlands, but results show several fold increases in CH_4_ emissions compared to drained conditions (Komulainen et al. 1998; Laudon et al. 2023; Tong et al. 2025), when GW level rose from well below the soil surface to close to it. This is to be expected as pristine peatlands also produce CH_4_. Because CH_4_ has a greater GWP over a 20-year timeframe (IPCC 2021), increasing CH_4_ emissions can counteract the positive effect of reduced soil CO_2_ emission after rewetting. However, large CH_4_ emissions may be avoided if rewetting results in a GW level just below (i.e., ~ 10 cm) the soil surface (Kasimir and Lindgren 2024).

The most potent GHG, N_2_O, with a 273 times higher GWP compared to CO_2_ (independent of time perspective due to its long atmospheric lifetime), has a complex mechanistic regulation. Key prerequisites for N_2_O emissions are available nitrogen and optimum oxygen content, with the latter being a function of the GW. Specifically, to start the process of N_2_O formation oxygen is required to allow nitrifiers to oxidize ammonia into nitrate. Small amounts of N_2_O may be released in this process. Large and sudden bursts of N_2_O can potentially occur if the process converting nitrate to nitrogen gas, in the anaerobic zone, is interrupted by access to oxygen. Thus, the GW level is also key to determine the potential for N_2_O emissions where fully water-saturated soils are expected to have low N_2_O emissions and a lowered GW level increases the risk of N_2_O production (Martikainen et al. 1993; Minkkinen et al. 2020). The soil carbon-to-nitrogen (C/N) ratio is a commonly used proxy for nutrient status, where a low ratio indicates nutrient-rich soils with high potential N_2_O emissions at low GW levels (Fig. 2c). In drained forested peatlands, N_2_O emissions have been shown to increase exponentially with decreasing C/N ratio (Klemedtsson et al. 2005; Mäkiranta et al. 2007), but the emission rates often show very large spatial and temporal variability (Maljanen et al. 2010; Leppelt et al. 2014; Tiemeyer et al. 2016).

The effect of peatland rewetting on dissolved C in stream runoff is another important factor to include in net climate warming estimates as aquatic fluxes can turn a peatland from an apparent C sink to a source under certain conditions (Leach et al. 2016). To date, there are only very few studies that have investigated runoff effects of DOC after peatland rewetting from boreal and hemiboreal conditions, and only recently the first DIC results following rewetting were reported (Zannella et al. 2024; Tong et al. 2025). Observed increase in stream DOC concentration immediately after rewetting has been found from both a nutrient-poor peatland in northern Sweden (Laudon et al. 2023; Tong et al. 2025) and nutrient-rich peatlands in Finland (Koskinen et al. 2011, 2017). More shallow GW levels, in combination with increased runoff water volumes, is the likely mechanism but how long lasting such enhanced export can be expected is not well established.

In essence, the purpose of rewetting for climate mitigation is to manage the water table such that the net CO_2_ sink-source strength is optimized without causing too large CH_4_ emissions and DOC leaching, while impacts on N_2_O balance also depend on soil fertility. Key in this context is to raise the GWL to just below the peat surface (i.e., ~ 10 cm) as a too low level will result in a continued C mineralization and CO_2_ emissions, while a too high level will provide more optimal conditions for CH_4_ production and emissions, as well as enhance the lateral C export via the downstream network. Hence, for a successful restoration of a specific peatland the establishment of the new water level will be the most critical aspect. This new GW should also need to be stable over time and hence not deteriorate because of erosion or degraded barriers, at the same time as it should provide a buffer for coping with future changes in the water balance.

## Timeframes for the development of climate benefit after rewetting

Rewetting has the potential to slow peat mineralization rates in both short- and long-term perspectives, but also restore biodiversity. The re-establishment of new GW levels after rewetting is in some sense simply a matter of refilling the drained pore space, which is key to halt the mineralization rate. A meta-analysis by Bring et al. (2022), focusing on GW effects of both ditch-cleaned and rewetted peatlands, suggested that the effects of the two management actions essentially had the same effect on the magnitude of hydrological alteration, but in opposite directions. The time it takes for a rewetting action to restore GW levels depends theoretically on the upstream catchment area in relation to the area being rewetted, in combination with the effectiveness of the ditch blocking. One example of this is the work by Karimi et al. (2024), who found that the initial recovery was rapid, taking only a few months for a new GW level to stabilize, but also that the new GW level became more sensitive to periods of low precipitation. As drained and managed peat soils have been compacted and the soil been partly mixed, the hydrological function can be irreversibly affected (Okruszko 1993; Menberu et al. 2021). Hence, a full re-establishment of the hydrological function may need a new peat layer to form on top of the affected peat before it is becomes fully functional.

Drainage activities close to one hundred years ago often resulted in altered environmental states that cannot immediately be reversed, because it might take years before natural peatland vegetation (Kreyling et al. 2021) and microorganisms re-establish, while aspects of the hydrological functioning (Menberu et al. 2021) may even take centuries to be fully re-gained. Hence, while a return to pristine conditions may never happen in some cases, a fundamental aspect in many rewetting ambitions is to restore key functions of natural peatlands. From a climate change mitigation perspective, the purpose is first to cease the ongoing GHG emissions that result from drainage. Secondly, the key is to re-establish the long-term GHG sink strength where the net CO_2_ uptake and storage rate exceeds the climatic impacts of vertical CH_4_ and N_2_O emissions, combined with the horizontal runoff losses of DOC and DIC. How far and fast the drained peat GHG emissions can be mitigated is generally unknown, but will likely be site specific as it depends on peat type, nutrient statues and upstream contributing catchment area.

**Fig. 3:**
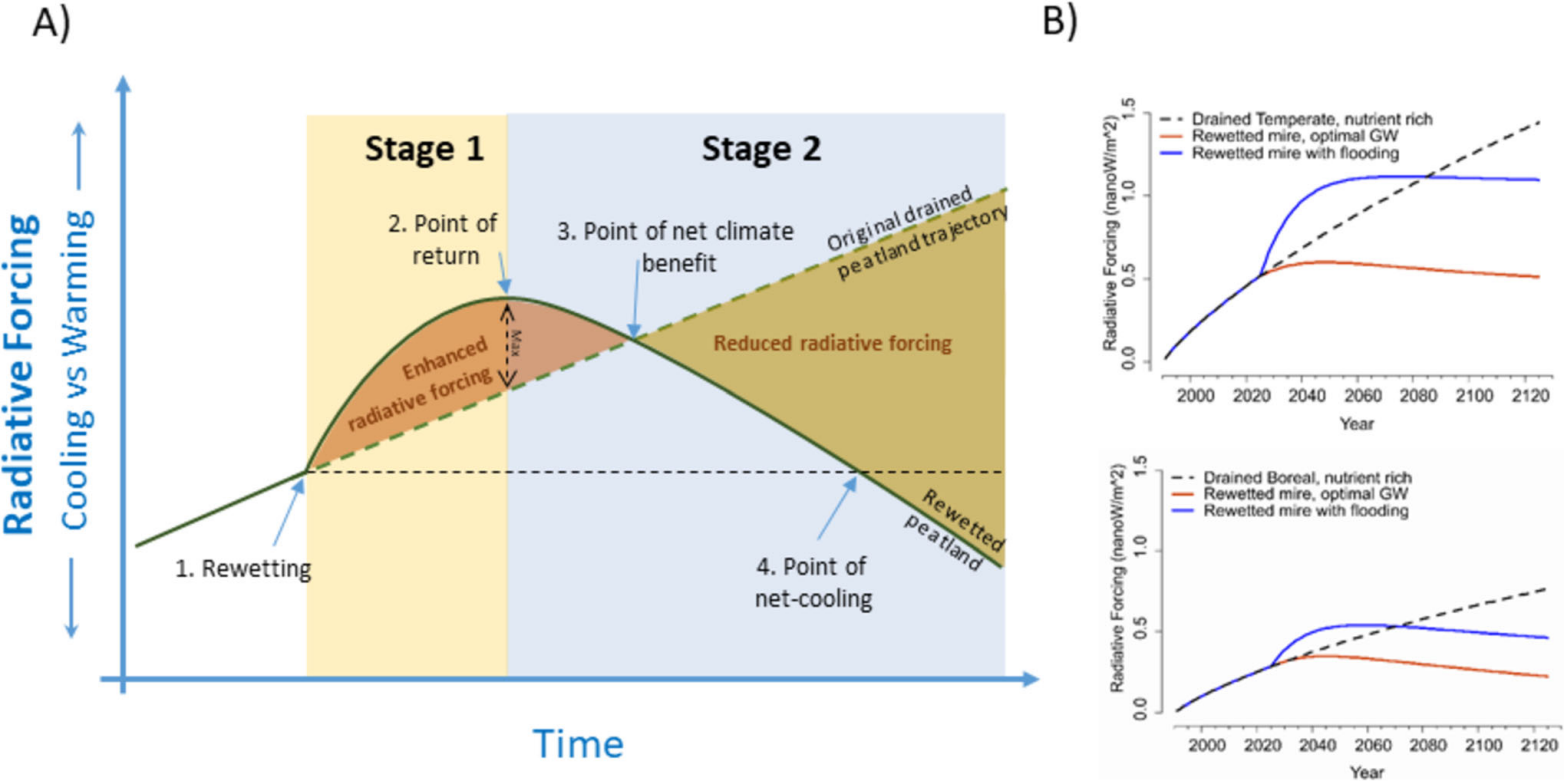
Panel **A**: A conceptual time line for rewetting effects on the radiative forcing over two distinct stages. Stage 1 is a period when the effect of increased CH_4_ emissions is larger than the effect of renewed CO_2_ sequestration and reduced emissions of N_2_O. This is most pronounced before the relative importance of CH_4_ is diminished due to its short atmospheric lifetime. Stage 2 occurs when the rewetted peatland starts the trajectory of becoming net-negative in a GHG balance perspective, and hence the effect of CO_2_ sequestration is larger than the climatic effect of the GHGs emitted to the atmosphere and exported via streams. Point 1. (point of *rewetting*) marks the timing of the restoration activities, point 2. (*point of return*) marks the transition from the initially enhanced net GHG impact to the beginning of decreasing net GHG impact, point 3. (*point of net climate benefit*) marks the time when the annual net GHG impact are back to the level of pre-rewetting conditions, and point 4. (*point of net-cooling*) marks the time when net-negative GHG impact are reached compared to the pre-rewetting conditions. Panel **B**—two modeling examples considering the soil only (based on Kasimir and Lindgren (2024)) of a temperate forestry-drained mire (upper) and boreal forestry-drained mire (lower) radiative forcing responses to optimized rewetting (− 30 to − 5 cm GW) versus flooding (> − 5 cm GW) effects. The model suggests that the peat soil under optimal conditions reach an immediate cooling effect, which is significantly larger for the nutrient-rich temperate peatland

Based on existing literature (e.g., Günther et al. 2020; Ojanen and Minkkinen 2020; Escobar et al. 2022; Kasimir and Lindgren 2024), we suggest that the development of the climate impact following rewetting can be functionally divided into two main stages with four key transitions points (Fig. 3). The first, Stage 1, follows as a direct consequence of rewetting, resulting in slowed peat mineralization rates, and hence reduced emissions of CO_2_, and in case of nutrient-rich soil conditions also of N_2_O. At the same time, increased CH_4_ emissions can be expected, combined with potentially enhanced stream C export. This first stage can result in enhanced peat soil GHG emissions, and hence even in climate warming, because of an increase in CH_4_ emissions. However, in the most nutrient-rich peatlands reduced N_2_O emission can potentially directly compensate, or at least shorten the negative Stage 1 effect of enhanced GHG emissions substantially.

The first point marks the *point of rewetting* action*.* The second is the *point of return* from an enhanced GHG impact (warming) after rewetting, to the beginning of a decreasing impact (cooling) (Fig. 3). This transition, which marks the start of Stage 2, occurs because the decreased soil CO_2_ (and N_2_O) emissions combined with vegetation CO_2_ uptake rate outweighs the effect of enhanced CH_4_ emissions and runoff C export that take off after rewetting, but also in part because the effect of elevated CH_4_ is reduced due to its relatively short atmospheric lifetime. As a result, the start of Stage 2 marks the trajectory phase toward climate cooling. Stage 2 is intercepted by two key points of transitions that are of importance; the *point of net climate benefit* occurs when the annual net climatic impact is back to the level of pre-rewetting conditions, and the *point of net-cooling* occurs when the climatic effect has reached below the starting point of the rewetting. It is important to note that this radiative forcing modeling complements the regular use of GWP, as the latter is not well suited to show the temporal dynamics of warming or cooling. The GWP method also struggles to describe the effect of the cumulative CO_2_ sequestration in relation to continued CH_4_ emissions, which is typical for peatlands.

The radiative forcing model shows the actual impact of rewetting over time on the radiative balance. It is helpful in the sense that it clearly distinguishes when and whether rewetting can be expected to achieve a desired outcome. The model suggests that the primary positive rewetting effect on the climate is from ceasing, or at least reducing the net emissions of CO_2_ and N_2_O during Stage 1, despite the increased CH_4_ emissions and losses through lateral C transport. It also suggests that for rewetting to be successful, there needs to be a strong plant related CO_2_ uptake that swiftly compensates for the new emission rates of CH_4_ (visible during Stage 2). Provided that the net balance of GHG impact primarily is regulated by the balance between peat mineralization rates, GHG emissions and soil C accumulation, the transformation into Stage 2 can be immediate as suggested by modeling, but could also take a few years to decades, or even centuries in some peatlands depending on system and GW level (Fig. 3b). That it can take several decades for most boreal rewetted peatlands before a *point of net-cooling* can be reached has been suggested by modeling (Ojanen and Minkkinen 2020, Launiainen et al. 2025), but it is also strongly dependent on the system boundary considered (Escobar et al. 2022). In the forested landscape, the system boundary becomes especially important as most modeling efforts do not include the trees, which in general are much more effective in CO_2_ uptake than any wet-adapted plants. Hence, the forest management strategy of existing trees of peatlands are a key area of uncertainty that needs careful consideration. Hence, the same system could directly reach the *point of net-cooling* after rewetting if only the soil is considered, or take decades to centuries if the trees, and therefore, the entire ecosystem is included.

Even if there is a risk that many rewetted peatlands temporarily will cause an increased warming after rewetting, this negative effect will be counteracted when the point of net climate benefit is reached (Fig. 3a). In contrast to forested peatlands, deeply drained agricultural peat soils with high CO_2_ and N_2_O emissions can despite increased CH_4_ emissions potentially reach the point of net climate benefit and net climate cooling more or less directly after rewetting (Kasimir and Lindgren 2024). Key in this context is also to appreciate that many peatlands that will not be rewetted can continue to be large CO_2_ emitters as long as there is peat left and that the only way to regain their stable C storage potential is to reduce mineralization rates by raising the GW level. In such cases, the faster rewetting efforts are put into action the sooner the peat degradation will be broken (Günther et al. 2020).

## Unanswered questions

Given the lack of empirical data from rewetted boreal and hemiboreal peatlands, several unknowns and uncertainties remain in order to fully understand and allow quantification of the climatic and biodiversity benefits and consequences. Key in this context is that each drained peatland has its own history, management strategy and land use and hence need site-specific management. Many unanswered questions are thus based on specific characteristics, whereas others are more universally unknown. While we in this work point out several know aspects, other questions warrant further studies.

### Hydrology key, but still not fully understood

Draining peatlands, and subsequently rewetting them a century later, is primarily a question of altering the GW level, which has fundamental impact on the ecology, biogeochemistry and microbiology, and therefore GHG balances of peatland soils. While it is well established that the mean GW level within a hydrological year often has reached back to a proximate pre-drained state, other key hydrological mechanisms remain uncertain. These include:

- How is rewetting affecting groundwater dynamics, *water retention characteristics and hydraulic conductivity*?
- How has peat degradation from mineralization and compaction affected the time it takes until full hydrological recovery.

### Rewetting effects on CO_2_ uptake and emission

While studies from rewetted peatlands in temperate regions have shown that it is possible to reach net-zero ecosystem CO_2_ emissions and even return to a CO_2_ sink function, variation among sites is high. Key questions that remain include:

- How do site-specific peatland properties affect post-rewetting CO_2_ trajectories, e.g., how does the response differ when rewetting a nutrient rich vs. poor, deep vs. moderately drained, etc.?
- How long will it take to establish wet-adapted species and particularly *Sphagnum spp.* in a boreal context, and when can a net annual CO_2_ sequestration be expected under different climatic and nutrient conditions?

### Rewetting effects on CH_4_ dynamics

The temporal evolution of CH_4_ post-rewetting is another unknown, with potentially high risks for large GHG emissions after rewetting. High emission of CH_4_ can be maintained for several decades when the GW level is at or above the soil surface as shown from rewetted peatlands in northern Germany (Vanselow-Algan et al. 2015; Kalhori et al. 2024), but the recorded effects from rewetting in boreal and hemiboreal regions are few. Specific questions include:

- How do site-specific properties affect CH_4_ emissions after rewetting?
- Is the knowledge gathered from pristine peatlands concerning CH_4_ emissions and plant dynamics also valid for rewetted conditions?
- What are the temporal, decadal dynamics of CH_4_ emissions after rewetting?

### Peatland N_2_O fluxes before and after rewetting

Variation in N_2_O emission is high, where the emission rate depends on nitrogen availability together with concomitant oxic and anoxic conditions. Fluctuating water level during the year may pose a risk of large N_2_O emissions (Leppelt et al. 2014), but also vegetation species may influence the risk (Eickenscheidt et al. 2014). On the other hand, vegetation free soil may also be a source of N_2_O, and from peat extraction sites, it has been suggested that rewetting can cause an overall reduction on N_2_O emissions (Järveoja et al. 2016; Jordan et al. 2016; Vybornova et al. 2019). Questions that need answers include:

- How effectively can peatland rewetting mitigate N_2_O emissions?
- Can the C/N ratio be used as a proxy for N_2_O emission size at both drained and rewetted peatlands, including both naturally nutrient rich and those previously used for agricultural purposes?

### Runoff effects on DOC and DIC as a rewetting consequence

While vertical exchange processes dominate most peatland rewetting studies, lateral losses of DOC and DIC remain a much less studied aspect of the overall climate benefit discourse. Losses of primarily DOC are important to consider as they can make up a significant portion of the net GHG balance of a boreal peatland (Nilsson et al. 2008) and drained peatland forests (Tong et al. 2024). Loss of DIC on the other hand is commonly believed to be less important because low pH results in lower concentrations of inorganic C being exported, although dissolved CH_4_ can also contribute to the overall budget (Leach et al. 2016). Specific unresolved questions include:

- Do factors such as nutrient level, treed vs treeless, climate region, former land-use etc. affect the response of DOC and DIC export to rewetting?
- What is the longevity of potential enhanced DOC and DIC concentration effects?

### The role of future forest management

Rewetting will preserve soil C but will likely reduce forestry output. In some peatlands, it is possible that altered forestry practices could become an alternative to rewetting (Laudon and Hasselquist 2023). This may be especially true if GWs are relatively high even with the forest cover, and if future ditch cleaning is not possible. Another alternative could be to rewet without removing all the standing biomass and evolve into practices such as continuous-cover forestry (Lehtonen et al. 2023) or longer rotation periods (Roberge et al. 2016) that could improve the C sequestration potential. Current practices of rewetting on boreal forested peatlands include harvesting the tree stems with roots, stumps and felling residues left on site. As a result, the tree CO_2_ uptake is eliminated, while the decaying logging residues contribute to CO_2_ emissions. Some of the urgent questions relate to:

- To optimize the rewetting effect is it better to leave or remove existing trees on a short- and long-term GHG balance perspective?
- Are there alternative forest management actions that are better for the minimizing the net GHG emissions at the time scale of the Paris agreement?

### Rewetting and climate change

In an era increasingly affected by climate change, we are now at risk of facing conditions that create the opposite obstacle to what the ditches solved—insufficient amounts of water—due to the expected increase in frequency, duration, and severity of drought. This may impact naturally wet peatlands changing them from net CO_2_ sinks to sources (Rinne et al. 2020). While this speaks for enhanced peatland rewetting efforts, better knowledge about how peatlands will respond to an altered climate remains critically important. From a climate change mitigation perspective, special concern should be devoted to:

- How do new climatic conditions affect the potential GHG benefits of rewetting?
- How will the re-establishment of higher GW levels increase the resilience to warmer and drier climate?

## Toward a better understanding of the climate impact of peatland rewetting

To improve empirical field-based understanding, there are three main approaches to such studies that may provide useful information: The first, and most powerful method, is to use field scale replicated and controlled experiments based on a so-called BACI-based approach (before-after and control impact). A BACI approach includes extended baseline measurements before the experimental treatment starts, followed by long-term monitoring of the post-treatment effect. In a Swedish context, to our knowledge only two ongoing studies apply a BACI designed experimental setup, both with so far relatively short time series. In Trollberget, a nutrient-poor mire in Northern Sweden, drained for foresty in the 1910s, was rewetted in the end of 2020 and followed since 2019 (Laudon et al. 2023), and in Skogaryd a nutrient-rich, deep-drained forested former agricultural peatland in Southwestern Sweden (Meyer et al. 2013) was clear-cut in 2020 and rewetted in 2022. Data have been published from the time the site was forested (Meyer et al. 2013). So far, only Trollberget has published data after rewetting (Laudon et al. 2023; Tong et al. 2025), but it is clear that these research infrastructures provide key information for understanding the effect on rewetting on the GHG balance required to begin deciphering the specific consequences of large-scale rewetting of Swedish peatlands. In order to be most useful for understanding rewetting effects, the monitoring has to continue for many years to come.

An alternative to long-term BACI designed studies is to use an approach called space-for-time substitution. Implicit for this method is to study specific effects of sites treated at different time points in the past. For this to work, the approach requires static climatic and other environmental conditions, consistent restoration treatments, and the same pre-treatment conditions. In the case of studying rewetting conditions, historically drained peatlands are used as controls, which are compared to those that were rewetted at the different time points, spanning from recently rewetted to those restored several decades ago. While the approach can be powerful, much of the problem in evaluating space-for-time substitution lies in assumption of constant climatic and environmental conditions. This assumption is likely always violated, because of nonstationary environmental and climatic conditions hardly ever exists. Despite the method limitations, the approach is commonly used and can provide useful information if interpreted carefully (Walker et al. 2010).

A third approach often used because of lack of data from rewetted forested peatlands is to use information from other peatland types or land uses, including both pristine and peat extraction sites. While there are much more empirical data available from both pristine boreal peatlands (Roulet et al. 2007; Nilsson et al. 2008; Maanavilja et al. 2011) and rewetted/restored peatland extraction sites (Tuittila et al. 1999, 2000; Waddington and Price 2000; Waddington and Day 2007; Strack and Zuback 2013; Järveoja et al. 2016), the comparison is problematic in several ways. While we can learn tremendously about natural processes from pristine peatlands, and they are key as reference sites for rewetting studies, it has been demonstrated that both hydrology and vegetation processes will take many decades to centuries before fully recovered (Kreyling et al. 2021). In contrast, peat extraction sites have the opposite problem, as a complete removal of several meters of the upper peat layers for commercial purposes make the comparison difficult. This is because new surfaces, made up by bare peat soils that can be many centuries to millennia old, lack any vegetation cover, have small functional seedbanks and hence may be regulated by entirely different dominant mechanisms.

In the quest to understand the effects and consequences of rewetting, empirical data are pivotal for predicting ecosystem response and function both under present and future climates. However, it will never be possible to measure all various effects and management approaches over the coming decades. Hence, improved process-based modeling for upscaling will continue to be important to reach better understanding (He et al. 2016; Kasimir et al. 2018). Novel methods of mapping using AI and high-resolution airborne laser scanning (ALS) for improving the mapping of peatlands (Ågren et al. 2022) and ditches (Busarello et al. 2025), combined with maps of historical land use (Ågren and Lin 2024), also have the potential to greatly improve our ability to identify the best sites for rewetting. By integrating site attributes into the design of new experiments from the outset, the relevance of these attributes can be systematically evaluated and improve both the models and our understanding of which landscape features are most critical for predicting rewetting outcomes, particularly as climatic and environmental conditions move toward states not previously experienced.

## Conclusions and outlook

Although much progress has been made the last decades on understanding the GHG effects of rewetting boreal and hemiboreal peatlands, the scientific field remains relatively immature with a number of unanswered key questions. While much work has been carried out on either relatively pristine peatlands or those that became drained a century ago, the main question is still what rewetting will do, and how long it will take for various functions to return to their natural state. Based on the present understanding, we provide a conceptual model, but also discuss some key steps of progress still in the need to be developed. Because of the large political interest and large investments made to rewet large peatland areas of Sweden, it is more essential than ever to start new rewetting studies that includes all aspects of GHG effect, hydrology, and biodiversity across the large climate and nutrient gradients that exist in Sweden. Furthermore, it is equally important to continue the few existing studies that already are ongoing as they can help answer questions about the long-term rewetting impact. However, while we wait for new empirical results, improved mechanistic understanding and better models, rewetting will continue. Hence, our ambition with this work is therefore to provide as much guidance as possible to any new or planned research on the topic for future generations.

## Data Availability

National available data used in the paper are available at the Swedish Forest Soil Inventory (SFSI).
