# Correction: Rewetting drained forested peatlands: A cornerstone of Sweden’s climate change mitigation strategy

**DOI:** 10.1007/s13280-025-02256-z

**Published:** 2025-09-27

**Authors:** Hjalmar Laudon, Järvi Järveoja, Anneli Ågren, Matthias Peichl, Amelie Lindgren

**Affiliations:** 1 Department of Forest Ecology and Management, Swedish University of Agricultural Sciences, 901 83 Umeå, Sweden; 2 Department of Earth Sciences, University of Gothenburg, 405 30 Gothenburg, Sweden

**Correction to: Ambio** 10.1007/s13280-025-02220-x

In this article Fig. 2 appeared as
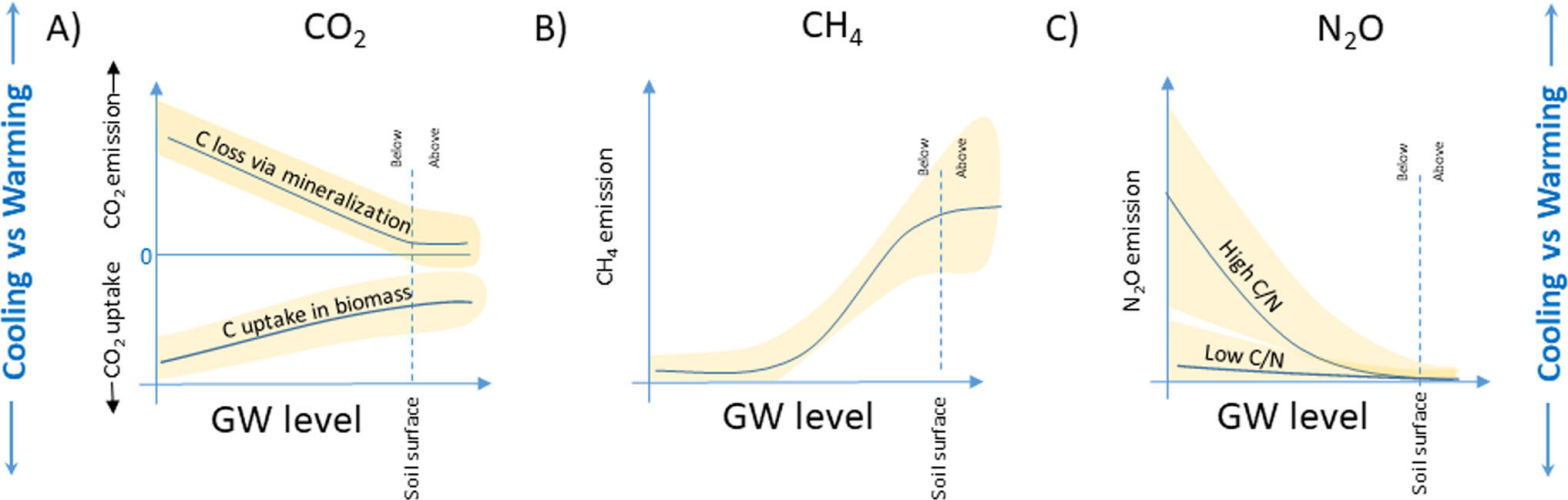


but should have appeared as

**Fig. 2:**
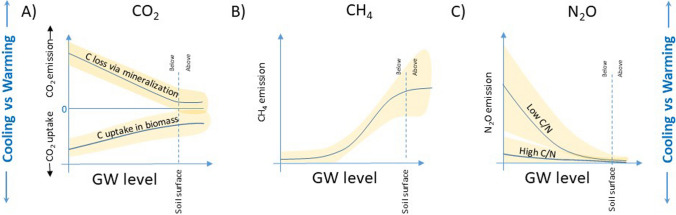
Groundwater (GW) level as a key regulator of greenhouse gas (GHG) emissions of carbon dioxide (CO_2_) (panel **A**), methane (CH_4_) (panel **B**), and nitrous oxide (N_2_O) (panel **C**). The yellow areas denote unexplained variability around the main effect

The original article has been corrected.

